# Unveiling Cathepsin B inhibition with repurposed drugs for anticancer and anti-Alzheimer’s drug discovery

**DOI:** 10.1371/journal.pone.0316010

**Published:** 2024-12-19

**Authors:** Mohammed Alrouji, Sabina Yasmin, Mohammed S. Alshammari, Fahad A. Alhumaydhi, Sharaf E. Sharaf, Moyad Shahwan, Anas Shamsi

**Affiliations:** 1 Department of Medical Laboratories, College of Applied Medical Sciences, Shaqra University, Shaqra, Saudi Arabia; 2 Department of Pharmaceutical Chemistry, College of Pharmacy, King Khalid University, Abha, Saudi Arabia; 3 Department of Clinical Laboratory Sciences, College of Applied Medical Sciences, Shaqra University, Shaqra, Saudi Arabia; 4 Department of Medical Laboratories, College of Applied Medical Sciences, Qassim University, Buraydah, Saudi Arabia; 5 Pharmaceutical Sciences Department, College of Pharmacy, Umm Al-Qura University, Makkah, Saudi Arabia; 6 Center of Medical and Bio-Allied Health Sciences Research (CMBHSR), Ajman University, Ajman, United Arab Emirates; University of Mashreq, IRAQ

## Abstract

Alzheimer’s disease (AD) is characterized by the aggregation of amyloid β (Aβ) peptides and the formation of plaques in the brain, primarily derived from the proteolytic degradation of amyloid precursor protein (APP). Cathepsin B (CatB) is a cysteine protease that plays a pivotal role in this process, making it a potential target for the development of anti-Alzheimer’s therapies. Apart from AD, CatB is implicated in various physiological and pathological processes, including cancer. Given the critical role of CatB in these diseases, identifying effective inhibitors is of significant therapeutic interest. In this study, we employed a systematic virtual screening approach using repurposed molecules from the DrugBank database to identify potential CatB inhibitors. Primarily, we focused on binding affinities and selectivity to pinpoint potential hits against CatB. Two repurposed molecules, Lurasidone and Paliperidone, emerged as promising candidates with significant affinity for CatB. These molecules demonstrated favorable drug profiles and exhibited preferential binding to the catalytic pocket of CatB via interacting with functionally significant residues. To further explore the binding mechanism and stability of the CatB-drug complexes, molecular dynamics (MD) simulations were conducted for 500 ns. The results revealed that CatB and Lurasidone, as well as Paliperidone, form stable complexes throughout the simulation. Taken together, the findings suggest that Lurasidone and Paliperidone can act as repurposed CatB inhibitors with potential applications in the development of therapeutics against AD and other CatB-associated diseases after further validation.

## 1. Introduction

Alzheimer’s disease (AD) remains a pressing global health challenge that impacts over 50 million individuals globally [1, 2]. It is characterized by a gradual decline in cognitive function and neuropathological changes, including the formation of amyloid β (Aβ) aggregates and plaques in the brain [3]. The proteolytic cleavage of amyloid precursor protein (APP) is a pivotal step in the generation of Aβ, and dysregulation of this process is considered a central pathological hallmark of AD [4]. Nonetheless, existing treatments for this condition offer only moderate efficacy and do not modify the trajectory of the neurodegenerative deterioration [5]. Neuroscientists worldwide are actively engaged in the development of a prospective medication for AD [6, 7]. One of the attractive targets for therapeutic intervention in the context of anti-Alzheimer’s drug discovery is Cathepsin B (CatB) [8, 9]. CatB is a lysosomal cysteine protease which belongs to the papain family and emerges as a key player in the proteolytic degradation of APP [10]. CatB is abundantly and ubiquitously expressed, unlike its more tightly regulated counterparts such as CatS or CatK [11].

CatB is a multifunctional protein that traditionally participates in various physiological processes such as wound healing, apoptosis, and the activation of thyroxine and renin [12]. Apart from this, research indicates that CatB plays a significant role in the onset and progression of various human diseases, including cancer and neurodegenerative disorders such as AD [13]. Additionally, upregulation of CatB has been observed in patients with rheumatoid arthritis [14] and inflammation [15]. Due to the multifaceted roles of CatB, targeting this enzyme holds potential not only for AD but also for cancer therapy [13]. In the cancer context, elevated activity of CatB is associated with increased tumor cell motility and invasiveness [16]. This elevated CatB activity correlates with poor therapy outcomes that emphasize its relevance as a promising therapeutic target for cancer as well [17]. Therefore, the use of CatB inhibitors has shown promise in reducing these malignant characteristics *in vitro* [18].

Repurposed drug discovery has emerged as an appealing approach in pharmaceutical research that offers a cost-effective and expedited route to identify new therapeutic uses for existing drugs [19]. The computational discovery of repurposed drugs has demonstrated significant value across various diseases, including neurodegenerative conditions [20]. Repurposed drugs hold immense promise as novel therapeutic agents [21]. By repurposing drugs originally developed for unrelated indications, this strategy holds immense promise for accelerating the translation of CatB-targeted therapies from bench to bedside. FDA-approved drugs, such as CA-074Me, E-64, and their analogues, have been extensively studied as CatB inhibitors for therapeutic interventions in AD and cancer [22, 23]. Synthesized molecules, including dipeptidyl nitriles and epoxysuccinyl derivatives, have demonstrated promising inhibitory activity against CatB *in vitro* and *in vivo* [24]. **S1 Fig** illustrates the chemical structures of CA-074Me, E-64, and selected synthesized molecules, highlighting their relevance as scaffolds for CatB inhibition. Here in this study, we aim to address the critical need for novel CatB inhibitors by conducting an integrated virtual screening of repurposed molecules from the DrugBank database. Our primary objective is to identify repurposed molecules that can inhibit CatB activity. In this study, we employed an *in silico* approach to screen the DrugBank database for repurposed drugs, aiming to assess their binding affinity with CatB.

## 2. Material and methods

### 2.1. Computer environment and web resources

Molecular docking-based virtual screening and visualization were conducted using multiple computational software, including MGL AutoDock tools [25], PyMOL [26], InstaDock [27], and Discovery Studio Visualizer [28]. These tools were used to explore ligand-receptor interactions and provide three-dimensional structural representations. Various bioinformatics resources such as UniProt [29], DrugBank [30], and Pass Online [31] were exploited for data retrieval and analysis. UniProt (https://www.uniprot.org/) and Protein Data Bank (PDB) (https://www.rcsb.org/) were served as useful resources for protein information, while the DrugBank database (https://go.drugbank.com/) used for pharmacological data on repurposed molecules. Additionally, Pass Online (https://www.way2drug.com/passonline/) was utilized to access information on biological activity and pharmacological profiles of the molecules.

### 2.2. Receptor and library preparation

The human CatB structure (accession number: 1GMY) was obtained from the RCSB website (http://www.rcsb.org) in PDB format [32]. The CatB structure underwent energy minimization and optimization before being employed in docking simulations. The selection of an optimal molecule library is crucial for enhancing the likelihood of identifying potential hits with efficacy. DrugBank is a freely accessible database that has an extensive collection of chemical structures and is suitable for structure-based virtual screening purposes [30]. In this study, we curated a library of repurposed drugs specifically from the DrugBank database that comprises ~3500 FDA-approved drug molecules. These molecules were checked for any inconsistencies and biologics and optimized for their structural configurations before docking studies.

### 2.3. Molecular docking-based virtual screening

Molecular docking-based virtual screening stands as a potent methodology within drug discovery [33]. Its primary function is to forecast the binding affinity and orientation of small molecular compounds with a target protein receptor. To identify high-affinity CatB-binding molecules, we employed a molecular docking screening approach. We utilize various bioinformatics tools, including MGL AutoDock tools, InstaDock, PyMOL, and Discovery Studio Visualizer, to conduct docking screening and interaction analyses. The processed CatB structure served as the target receptor, while the DrugBank database of repurposed drugs was utilized as a source of ligands. InstaDock v1.2 was used for the docking process while employing a blind search space with dimensions of 61 Å, 64 Å, and 60 Å in the X, Y, and Z axes, respectively. The search space was centralized at coordinates 19.881 Å, 37.74 Å, and 37.191 Å. The docking protocol was validated using a redocking approach, a commonly employed method in molecular docking research. In this procedure, a dipeptidyl nitrile inhibitor was docked back into CatB, and the resulting docked pose was compared to its original co-crystallized structure. After the docking calculations, the top hits were identified on the basis of their docking scores. All the probable docked conformers were splitted for subsequent analysis of the interaction mechanism.

### 2.4. Protein-ligand interactions and PASS analysis

A comprehensive protein-ligand interaction analysis was conducted to elucidate the binding patterns between proteins and ligands. PyMOL and Discovery Studio Visualizer played a crucial role in studying the binding prototype of the screened molecules and the CatB binding site. The software allowed for a detailed exploration of potential molecular interactions. The optimal donor-acceptor distance for hydrogen bonds in protein-ligand complexes was considered between 2.5–3.5 Å. The optimal hydrogen bond angle (donor-hydrogen-acceptor angle) were considered between 135–180°. The biological properties of the screened molecules were predicted using the PASS website. The PASS server works based on the structural-activity relationship of a molecule to predict its potential biological and pharmacological properties [31]. The PASS algorithm compares the molecular structure to an in-house dataset encompassing diverse biological functions to make predictions. The predictive analysis is based on the probability to be active (Pa) and probability to be inactive (Pi). A higher Pa value signifies a greater likelihood of a specific biological activity for the molecule under investigation.

### 2.5. Molecular dynamics simulations

MD simulations of proteins are useful for studying their dynamic behavior at an atomic level over time [34]. These simulations deliver insights into the structural changes, conformational dynamics, and binding mechanisms of protein-ligand complexes, which are vital for understanding their biological function and drug discovery efforts [35]. We have conducted MD simulations using the GROMACS 2020 beta suite [36] on an HP Z840 computer. The simulations involved the CatB protein in both its free and ligand-bound forms, interacting with Lurasidone, Paliperidone, and Ca-074Me. The crystal structure of CatB and the selected ligands were obtained from the docking study and prepared for MD simulations. The CHARMM36-JUL2022 force field [37] was employed for the simulation procedure. To facilitate the simulations, topology parameters for Lurasidone and Paliperidone were generated using the PRODRG website [38]. Each complex was placed within a cubic boundary box with a 10Å radius. The TIP3P solvent model was utilized for solvation in aqueous environments [39]. To maintain system neutrality, an adequate number of counter ions were supplied. Energy minimization was performed using the steepest descent algorithm to remove steric clashes and achieve a maximum force below 1000 kJ/mol/nm. The system was then equilibrated in two phases: first under an NVT ensemble (constant Number of particles, Volume, and Temperature) for 100 ps at 300 K, followed by an NPT ensemble (constant Number of particles, Pressure, and Temperature) for an additional 100 ps at 1 bar. In both equilibration phases, heavy atoms of the protein backbone and ligand were restrained with a force constant of 1000 kJ/mol/nm^2^, allowing the solvent and ions to equilibrate around the protein and protein-ligand complex. Subsequently, a 500 ns run was executed for CartB and its complexes with the screened molecules. The integration time step was set to 2 fs, and the LINCS algorithm was used to constrain bond lengths. Long-range electrostatic interactions were computed using the Particle Mesh Ewald (PME) method, with a cutoff of 1.0 nm for both van der Waals and Coulomb interactions. Trajectory data were saved every 10 ps for subsequent analysis. The resulting trajectories were analyzed using the integrated tools provided by GROMACS, which enabled a thorough examination of the dynamic behavior and interactions within the simulated systems.

### 2.6. Principal component and free energy landscape analyses

To unravel fundamental protein motions and explore folding kinetics, principal component analysis (PCA) was employed. [40]. It utilizes an essential dynamics approach for the conformational mapping of simulated trajectories [41]. This technique facilitated the examination of conformational sampling in CatB and its complexes with Lurasidone, Paliperidone, and Ca-074Me by diagonalizing the eigenvectors (EVs) for the covariance matrix. Through this approach, we gained insights into the essential motions and dynamics of the studied systems. Additionally, free energy landscape (FEL) analysis [40] was conducted on the simulation trajectories generated. This analysis is useful to explore the folding behavior of CatB in both its free and ligand-bound states, interacting with Lurasidone, Paliperidone, and Ca-074Me. This analysis provided a detailed insight into the energy landscapes governing the structural transitions and stability of the systems that shed light on the potential impact of ligand binding on the protein’s conformational dynamics.

### 2.7. MM-PBSA calculation

To estimate the binding free energy of the protein-ligand complexes, the Molecular Mechanics Poisson-Boltzmann Surface Area (MM-PBSA) approach was used [42]. This approach is a useful asset in drug discovery to evaluate the stability of molecular interactions. The calculation employed data from MD simulations, a 10 ns frame selected from the stable part of the MD trajectory, i.e., 300–310 ns. MM-PBSA calculations for each protein-ligand complex were done using the gmx mmpbsa module in GROMACS software [43]. This tool uses the following equation to calculate the binding energy of the protein-ligand complex:

$$\Delta G_{Binding}=G_{Complex}-(G_{Protein}+G_{Ligand})$$

where *G*_Complex_ represents the total free energy of the binding complex, and *G*_Protein_ and *G*_Ligand_ are the measure of total free energies of KatB and the compounds Lurasidone, Paliperidone, and Ca-074Me, respectively.

## 3. Results and discussion

### 3.1. Molecular docking-based virtual screening

Molecular docking-based virtual screening is a powerful tool in drug discovery that allows rapidly screen large chemical libraries and prioritize the most promising candidates for the drug discovery process [44]. To identify high-affinity binding partners for CatB, a molecular docking-based virtual screening was conducted on a library comprising 3500 molecules sourced from the DrugBank database. Utilizing InstaDock, each molecule underwent molecular docking screening, which generates affinity scores and docked poses. The results of the docking protocol validation confirmed its ability to accurately replicate the binding pose of a dipeptidyl nitrile inhibitor from the CatB co-crystal structure (PDB ID: 1GMY). The alignment of the docked pose with the co-crystallized structure demonstrated their close correspondence (**S2 Fig**). This validation highlights the reliability of the docking methodology and its effectiveness in analyzing molecular interactions and ligand binding. To enhance the efficiency of discovering novel CatB inhibitors, the binding affinity emerged as a crucial filter during the screening process. The top 10 molecules were selected based on their projected binding affinity scores with CatB. The results revealed that these chosen molecules showed noteworthy docking scores, ranging from −8.7 kcal/mol to −10.6 kcal/mol (**Table 1**). Notably, all of these molecules demonstrated higher binding affinity for the CatB than the reference inhibitor Ca-074Me [45]. This screening narrowed down the selection of molecules and highlighted a few candidates with noteworthy affinities against CatB. This will lay the foundation for the subsequent stages of analysis of promising molecules for further exploration as potential CatB modulators.

**Table 1:** The top 10 hits based on their docking scores towards CatB.

S. No.	Drug	Affinity (kcal/mol)	pKi	Ligand Efficiency (kcal/mol/non-H atom)	Torsional Energy
1.	Bisdequalinium	−10.6	7.77	0.2409	0
2.	Conivaptan	−9.8	7.19	0.2579	1.2452
3.	Nilotinib	−9.6	7.04	0.2462	2.1791
4.	Radotinib	−9.4	6.89	0.241	2.1791
5.	Perlutal	−9.2	6.75	0.2788	0.6226
6.	Pimozide	−9.1	6.67	0.2676	2.1791
7.	Bictegravir	−9.0	6.6	0.2812	1.2452
8.	Lurasidone	−8.8	6.45	0.2514	1.5565
9.	Paliperidone	−8.8	6.45	0.2839	1.5565
10.	Aprepitant	−8.7	6.38	0.2351	2.4904
11.	Ca-074Me	−6.5	4.77	0.2321	3.113

### 3.2. Drug profiling in PASS analysis

The PASS server is instrumental in predicting the biological activity of small molecules [31]. In our drug profiling analysis, the PASS server was utilized to explore the biological activity of screened molecules from the docking screening. Here, two molecules, Lurasidone and Paliperidone passed the PASS biological activity screening that showcase their potential therapeutic attributes related to AD treatment. Notably, the findings indicate that these molecules possess AD treatment, cognition disorders treatment, antineurotic, antipsychotic, nootropic, dementia treatment, mood disorders treatment, antidepressant, and neurodegenerative disease treatment capabilities. The PASS analysis revealed high probabilities for expected biological properties with Pa varying from 0,126 to 0,946 (**Table 2**). The outcomes provide valuable insights into the potential pharmacological effects of Lurasidone and Paliperidone, specifically in the realms of anti-AD management. These results are crucial for prioritizing and further investigating these molecules in subsequent stages of drug repurposing.

**Table 2:** The top 10 relevant PASS predictions of the screened molecules.

Molecule	Pa	Pi	Biological Activity
Lurasidone	0,933	0,004	Antipsychotic
	0,548	0,005	Antiadrenergic
	0,484	0,027	Anxiolytic
	0,354	0,089	Polarisation stimulant
	0,278	0,076	Alzheimer’s disease treatment
	0,280	0,082	Cognition disorders treatment
	0,347	0,202	Antineurotic
	0,219	0,093	Cardiovascular analeptic
	0,160	0,043	Sepsis treatment
	0,126	0,009	Dysmenorrhea treatment
Paliperidone	0,946	0,003	Antineurotic
	0,872	0,004	Antipsychotic
	0,815	0,015	Nootropic
	0,475	0,003	Bipolar disorder treatment
	0,378	0,046	Anxiolytic
	0,348	0,090	Dementia treatment
	0,261	0,037	Dependence treatment
	0,279	0,068	Mood disorders treatment
	0,269	0,070	Antidepressant
	0,279	0,157	Neurodegenerative diseases treatment
Ca-074Me	0,900	0,006	Nootropic
	0,562	0,008	Neuropeptide Y4 antagonist
	0,487	0,114	Antiischemic, cerebral
	0,368	0,004	Neurotrophic factor
	0,275	0,008	Neurotensin 1 receptor agonist
	0,227	0,013	Neurolysin inhibitor
	0,267	0,060	Calpain inhibitor
	0,203	0,009	Cathepsin L inhibitor
	0,174	0,005	CatB inhibitor
	0,229	0,117	Alzheimer’s disease treatment

Pa, probability to be active; Pi, probability to be active

### 3.3. Interaction analysis

**Fig 1:**
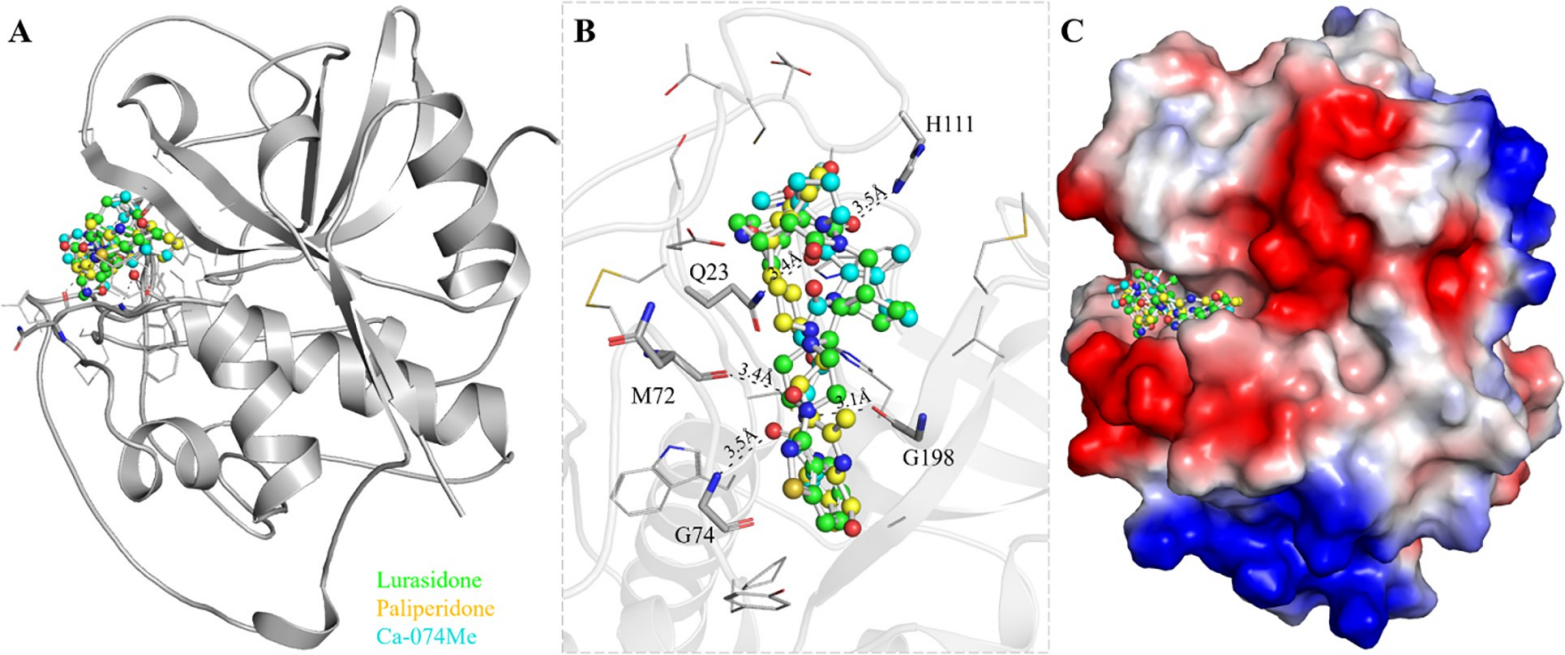
CatB in complex with the screened molecules. (A) Cartoon view of CatB with Lurasidone (green), Paliperidone (yellow), and Ca-07Me (cyan). (B) Magnified cartoon view of CatB interacting residues with the elucidated molecules. (C) Charged view of CatB binding pocket filled by the elucidated molecules.

In the CatB active site, the S′ pocket is architected with unique features among papain superfamily proteases, which makes it crucial for designing CatB inhibitors [11]. Unlike other Cat proteases such as CatL, S, and K, which have quite open S′ regions, the S′ region of CatB is obstructed by a 19-residue insertion [11]. Within this insertion, His110 and His111 have previously demonstrated an affinity for binding carboxylate groups, thereby imparting CatB with its distinctive C-terminal dipeptidyl exopeptidase activity [46]. Exploiting this binding pocket, we aimed to explore the probable binding interactions between the elucidated molecules, Lurasidone and Paliperidone. The analysis showed that multiple docked conformers of Lurasidone and Paliperidone were observed to engage with specific residues in a mutually interactive manner (**Fig 1**). The analysis revealed several key hydrogen bonding interactions between the compound and the protein binding site. Notably, the analysis revealed direct hydrogen bonding interactions with important residues of the CatB binding site, including Gln23, Met72, Gly74, His11, and Gly198 (**Fig 1A**). The binding prototypes of Lurasidone and Paliperidone showcased shared interactions with the important residues of the CatB binding pocket (**Fig 1B**). Gln23, Met72, Gly74, His111, and Gly198 form hydrogen bonds with Paliperidone with a distance of 3.4Å, 3.4Å, 3.5Å, 3.5Å, and 3.1Å, respectively. All the hydrogen bonds in protein-ligand complexes were formed between 3.0–3.5 Å. Here, the donor-hydrogen-acceptor angles were calculated between 135–180° for all the bonds. The molecules are intricately layered on each other, forming direct contact with the CatB active site in close proximity.

Interestingly, both molecules were observed to dock together, effectively obstructing CatB’s binding site while fitting closely into the deep cavity (**Fig 1C**). Both molecules demonstrated several common interactions with CatB as the reference inhibitor Ca-074Me. This interaction analysis provides a structural understanding of how Lurasidone and Paliperidone bind to CatB which underscores their potential to collectively interfere with the binding site. The structure-activity relationship (SAR) based on binding score analysis revealed that Lurasidone and Paliperidone exhibited stronger binding to CatB due to their ability to form multiple hydrogen bonds with key residues alongside hydrophobic interactions within the catalytic pocket. Lurasidone’s sulfonamide and piperazine moieties facilitated π-π stacking and hydrophobic contacts, while Paliperidone’s hydroxyl and ketone groups contributed to hydrogen bonding. In contrast, CA-074Me showed fewer interactions, correlating with its lower binding score. These findings indicate that functional group modifications, such as adding hydrophobic substituents near the catalytic site, could further enhance binding affinity and selectivity. These insights into the molecular interactions contribute significantly to the characterization of the binding mechanism and guide the exploration of these molecules as potential CatB inhibitors.

**Fig 2:**
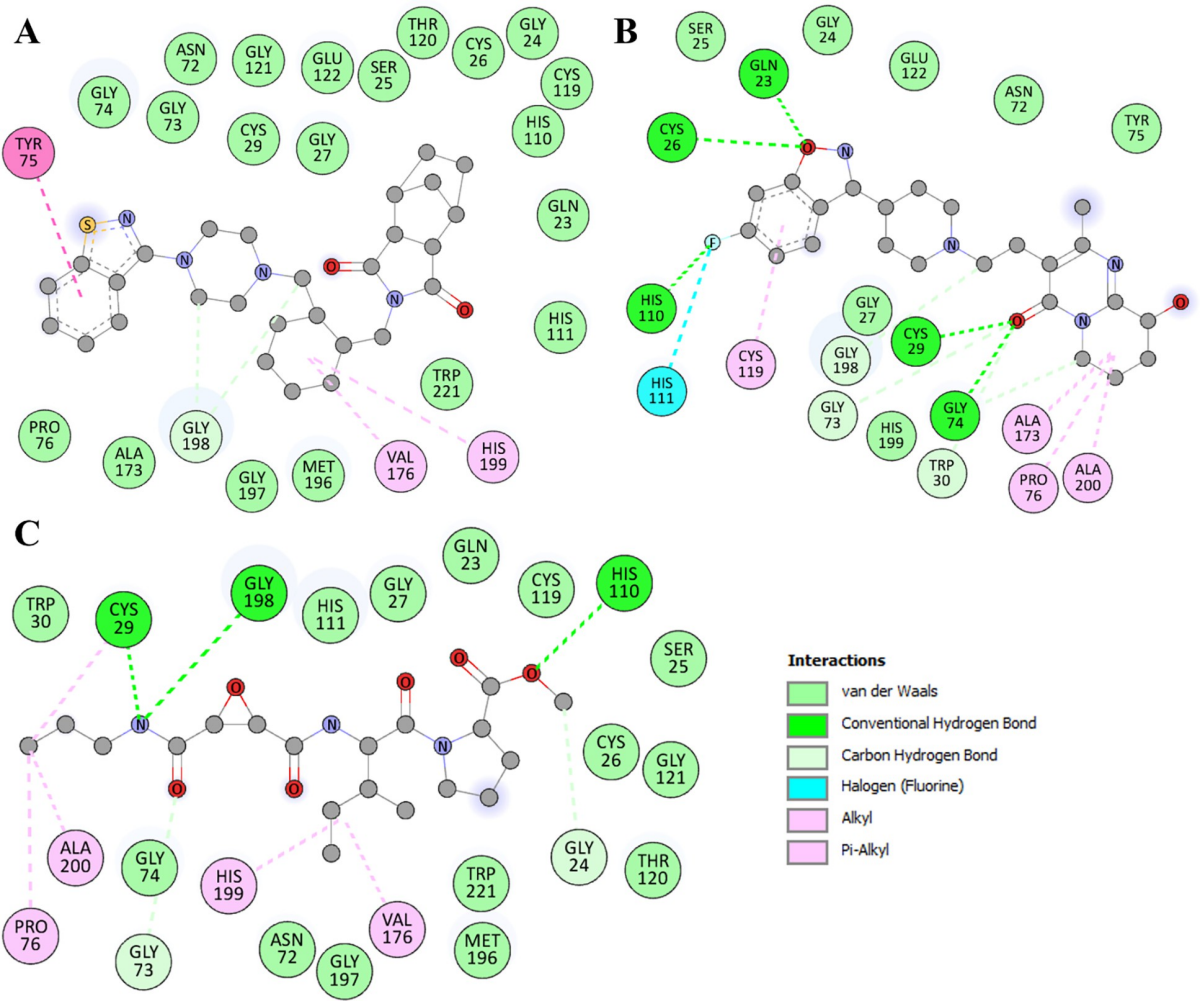
The two-dimensional representation of CatB residues and their interaction with **(A)** Lurasidone, **(B)** Paliperidone, and **(C)** Ca-074Me.

A detailed exploration of non-covalent interactions is crucial for deciphering the binding characteristics and nature of molecular associations. Detailed interaction analyses were conducted using Discovery Studio Visualizer to delve into the binding prototype and interaction patterns of the screened molecules (**Fig 2**). The study revealed various close interactions between Lurasidone and Paliperidone with specific residues (**Table 3**). For Lurasidone, hydrogen bonding was observed with Gly198, while several other residues participated in various additional interactions, i.e., Tyr75, Val176, and His199 (**Fig 2A**). It also showed several Van der Waals interactions with Gln23, Gly24, Ser25, Cys26, Gly27, Cys29, Asn72, Gly73, Gly74, Tyr75, Thr120, Gly121, Glu122, Cys119, Pro76, His110, His111, Gly197, Met196, and Trp221. Simultaneously, Paliperidone exhibited hydrogen bonding interactions with Gln23, Cys26, Cys29, Gly74, and His110, with further involvement in various hydrophobic interactions with other residues such as Pro76, His111, Cys119, Ala173, and Ala200 (**Fig 2B**). It also showed several Van der Waals interactions with Gly24, Ser25, Gly27, Tyr75, Glu122, and His199. Both molecules displayed multiple interactions as the reference inhibitor Ca-07Me with CatB’s binding site residues important for its functional activity (**Fig 2C**). These interactions signify strong and specific associations between Lurasidone and Paliperidone with key CatB residues. Importantly, these interactions within the binding pocket suggest that the stability of Lurasidone and Paliperidone may impede CatB’s access to its binding site, consequently leading to the functional inhibition of CatB. This molecular insight highlighted the potential inhibitory mechanisms of Lurasidone and Paliperidone and underscored their promise as candidates for repurposed CatB inhibitors.

**Table 3:** Various interaction types and their corresponding residues involved in binding CatB with the identified compounds.

S. No.	Compound	Affinity (kcal/mol)	Interaction			
			Hydrogen bonds	Alkyl/Pi-interactions	Van der Waals	Other
	Lurasidone	−8.8	Gly198	Tyr75, Val176, His199	Gln23, Gly24, Ser25, Cys26, Gly27, Cys29, Asn72, Gly73, Gly74, Tyr75, Thr120, Gly121, Glu122, Cys119, Pro76, His110, His111, Gly197, Met196, Trp221	
	Paliperidone	−8.8	Gln23, Cys26, Cys29, Trp30, His110, Gly73, Gly74, Gly198	Pro76, Cys119, Ala173, Ala200	Gly24, Ser25, Gly27, Tyr75, Glu122, His199	His111
	Ca-074Me	−6.5	Gly24, Cys29, Gly73, His110, Gly198	Pro76, Val176, His199, Ala200	Gln23, Ser25, Cys26, Gly27, Trp30, Gly73, Gly74, Thr120, Gly121, Gly197, Trp221	

In addition to repurposing Lurasidone and Paliperidone, further modifications of the structures can be made to increase the biological activities of these compounds. New functional groups at specific positions of the ligand structures would improve the hydrogen bonding with the essential residues such as His111 and Gly198, which are responsible for the stability of the ligand in the active site. The presence of methyl or ethyl groups near the catalytic pocket could improve the interaction with the hydrophobic regions of the CatB active site, which may enhance the binding constant. The conformational flexibility of the ligands could be minimized by replacing the flexible bonds with ring systems or linkers. This would help maintain the binding pose in a more favorable conformation through the entire interaction with CatB, which may help stabilize the complex and increase inhibition. These modifications can provide useful data for the additional lead optimization to increase the CatB inhibitory activity of Lurasidone and Paliperidone. Further experimentation and fine-tuning of these changes may lead to the development of better inhibitors of CatB.

### 3.4. Molecular dynamics simulations

MD simulations on docked complexes play a vital role in refining docked models by considering the inherent flexibility of proteins and protein-ligand interactions [47]. MD simulations contribute to elucidating structural details and the conformational behavior of proteins and protein-ligand systems [34]. In this study, MD simulations were conducted on CatB and its bound states with Lurasidone, Paliperidone, and Ca-074Me in the TIP3P solvent environment using a charmm36-jul2022 force field. The goal was to examine the conformational landscape and stability of the protein-ligand systems throughout 500 ns. Utilizing the best poses from the docking results, CatB complexes with Lurasidone, Paliperidone, and Ca-074Me were subjected to a 500 ns MD simulation. The time evolution of different factors was systematically examined to gain a detailed insight into the structural changes and flexibility of CatB over the simulation period, as discussed in the ensuing sections.

#### 3.4.1. Structural changes and compactness

**Fig 3:**
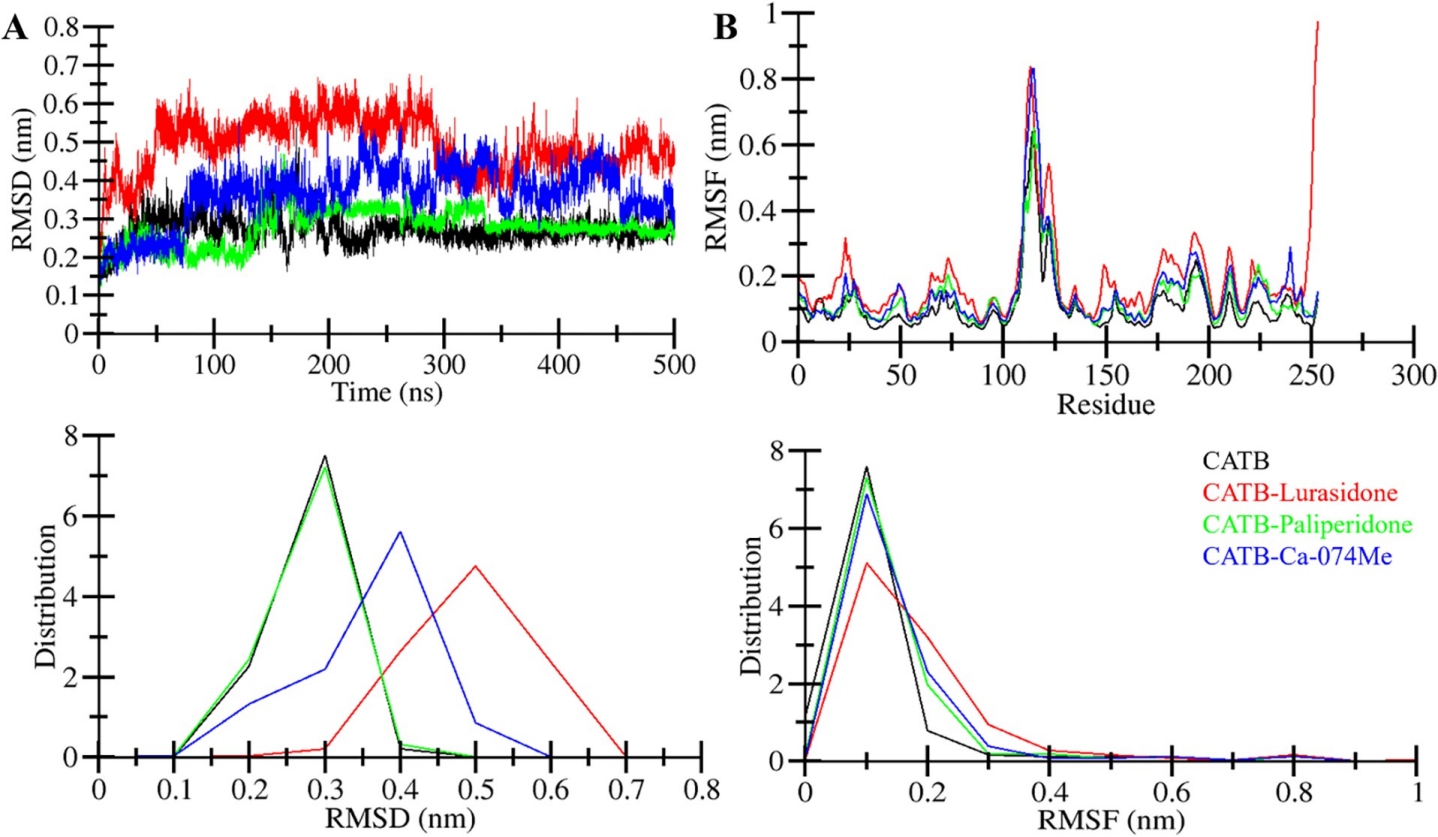
Structural dynamics of CatB upon Lurasidone, Paliperidone, and Ca-074Me binding. (**A**) RMSD plot of CatB in complex with Lurasidone and Paliperidone. (**B**) RMSF plot of CatB and its complex with Lurasidone and Paliperidone. The lower panels illustrate the distribution of the values as a probability distribution function (PDF).

Root-mean-square deviations (RMSD) analysis is a useful tool for assessing structural variations in protein over time [48]. In this study, we utilized RMSD and RMSF analyses to evaluate the backbone deviations of CatB in both the CatB-Lurasidone, CatB-Paliperidone, and CatB-Ca-074Me complexes during a 500 ns MD simulation. The RMSD data generated from the simulation trajectory were employed to construct time evolution charts illustrating backbone aberrations (**Fig 3A**). Notably, the plot revealed random variations in RMSD up to 300, reaching ~0.3 nm to 0.6 nm, especially in CATB-Lurasidone and CATB-Ca-074Me complexes (**Fig 3A, upper panel**). Despite these early fluctuations, the stability of all four systems became evident through constant and modest oscillations in the backbone atoms without any major deviations. At the same time, CATB-Paliperidone shows quite stability throughout the simulations. The probability distribution function (PDF) plot also indicates higher stability of the CATB-Paliperidone complex (**Fig 3A, lower panel**).

To further elucidate the dynamics of CatB at the residue level, we examined the root mean square fluctuations (RMSF) in all four systems. The result shows a similar distribution of RMSFs for all the systems, with some increased dynamics in the protein-ligand complexes. CATB-Lurasidone shows higher residual fluctuations than CATB, CATB-Paliperidone, and CATB-Ca-074Me systems (**Fig 3B, upper panel**). The PDF representation demonstrated similar RMSF distribution with reduced probability, particularly in the ligand-bound states, indicating the stability of these systems with minimal structural deviation during the simulation (**Fig 3B, lower panel**). Taken together, the RMSD and RMSF analyses collectively indicate that ligand-bound systems, particularly the CatB-Paliperidone complex, are stable and do not undergo significant structural deviations during the 500 ns simulation period.

**Fig 4:**
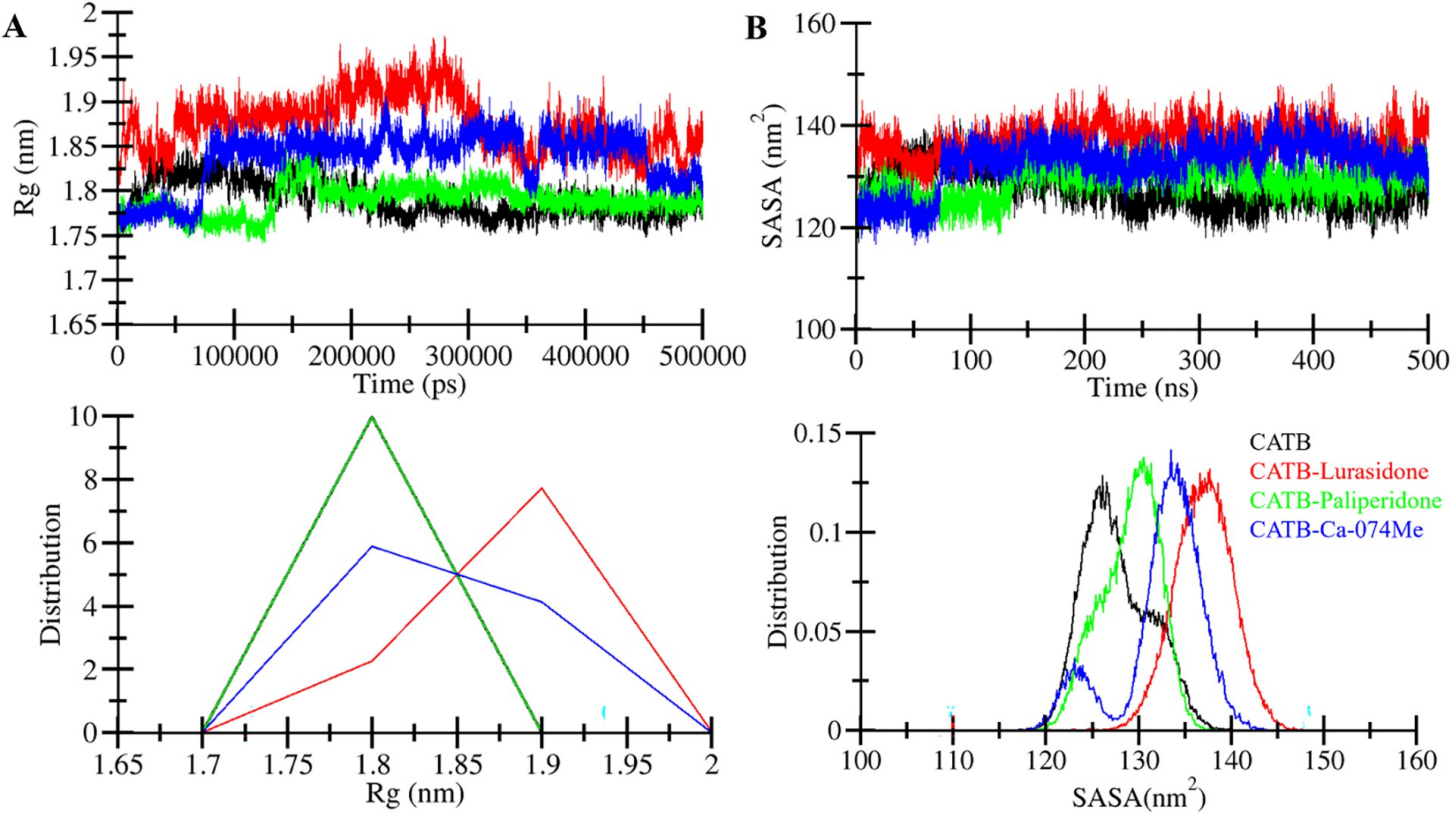
Structural compactness of CatB before and after Lurasidone, Paliperidone, and Ca-074Me binding. (A) The radius of gyration plot and (B) Solvent-accessibility plot of CatB with Lurasidone, Paliperidone, and Ca-074Me binding.

The radius of gyration (*R*g) is a widely used metric to assess protein compactness and structural dynamics [49]. In this study, the temporal evolution of *R*g was employed to investigate the compactness of CatB in both its free and ligand-bound states (CatB-Lurasidone, CatB-Paliperidone, and CatB-Ca-074Me) throughout the 500 ns simulation (**Fig 4A**). The *R*g plot illustrated that CatB, in the presence of Paliperidone, maintained a steady range between 1.75 nm and 1.85 nm throughout the simulation (**Fig 4A, upper panel**). The slight increase in folding dynamics observed after Lurasidone binding did not compromise the overall stability of CatB. The PDF plot further corroborated these findings, showing that *R*g values for CatB, while slightly increased after Lurasidone binding, remained within a consistent range (**Fig 4A, lower panel**).

Solvent-accessible surface area (SASA) analysis describes the area of a molecule surrounding its neighboring solvent [50]. SASA is crucial for understanding protein folding in solvent conditions [51]. The time evolution of CatB’s SASA, both before and after Lurasidone and Paliperidone binding, demonstrated a slight increment but no significant changes in SASA values throughout the simulation (**Fig 4B**). The SASA analysis suggested that the CatB structure remained stable even in the presence of Lurasidone, Paliperidone, and Ca-074Me (**Fig 4B, upper panel**). The PDF plot also exhibited slightly increased SASA values for CatB when complexed with Lurasidone, Paliperidone, and Ca-074Me (**Fig 4B, lower panel**). While total SASA measures the exposure of the entire complex to solvent, buried SASA (burSASA) is used to evaluate the stability of the binding interface between a protein and its ligand. Buried SASA is calculated by subtracting the sum of the individual SASA values of the protein and ligand from the SASA of the complex. The burSASA values were 2.69 nm^2^, 2.82 nm^2^, and 2.22 nm^2^, respectively, indicating Paliperidone forms the most stable interaction with CatB. A higher burSASA suggests a stronger protein-ligand binding interface, with Paliperidone showing the greatest buried surface area, followed by Ca-074Me and Lurasidone. This analysis highlights Paliperidone and Lurasidone’s potential as effective inhibitors based on their stable binding. In summary, the *R*g and SASA analyses collectively indicate that CatB remains structurally stable and compact throughout the 500 ns simulation in both its free and ligand-bound forms. The analysis offered valuable insights into the protein’s dynamic nature and interactions with Lurasidone and Paliperidone.

#### 3.4.2. Dynamics of intramolecular hydrogen bonding

**Fig 5:**
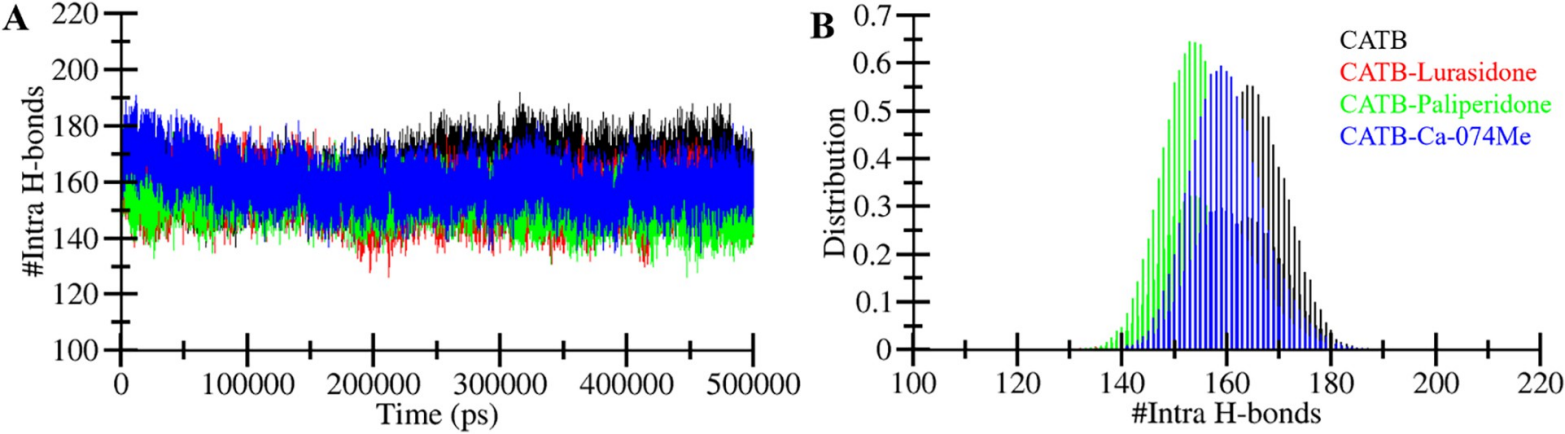
Dynamics of hydrogen bonds. (**A**) Hydrogen bonding in CatB, before and after Lurasidone, Paliperidone, and Ca-074Me binding. (**B**) The probability density function (PDF) of the hydrogen bond numbers.

Intramolecular hydrogen bonding plays a crucial role in stabilizing the structural conformation and stability of a protein molecule [52]. In this study, we analyzed the number of hydrogen bonds formed within CatB over time using MD trajectories. This analysis allowed us to assess the stability of hydrogen bonding in CatB before and after the binding of Lurasidone and Paliperidone. The results illustrate fluctuations in the number of hydrogen bonds within CatB, both before and after Lurasidone and Paliperidone binding (**Fig 5A**). The graph indicates that the hydrogen bonds formed within CatB were persistent and contributed to the stability of its protein structure. A minor decrement in the hydrogen bonds within the CatB-Lurasidone, CatB-Paliperidone, and CATB-Ca-074Me complexes was observed. This decrease may arise from the utilization of some intramolecular space by the molecules, as revealed in the PDF plot (**Fig 5B**).

#### 3.4.3. Dynamics of intermolecular hydrogen bonding

**Fig 6:**
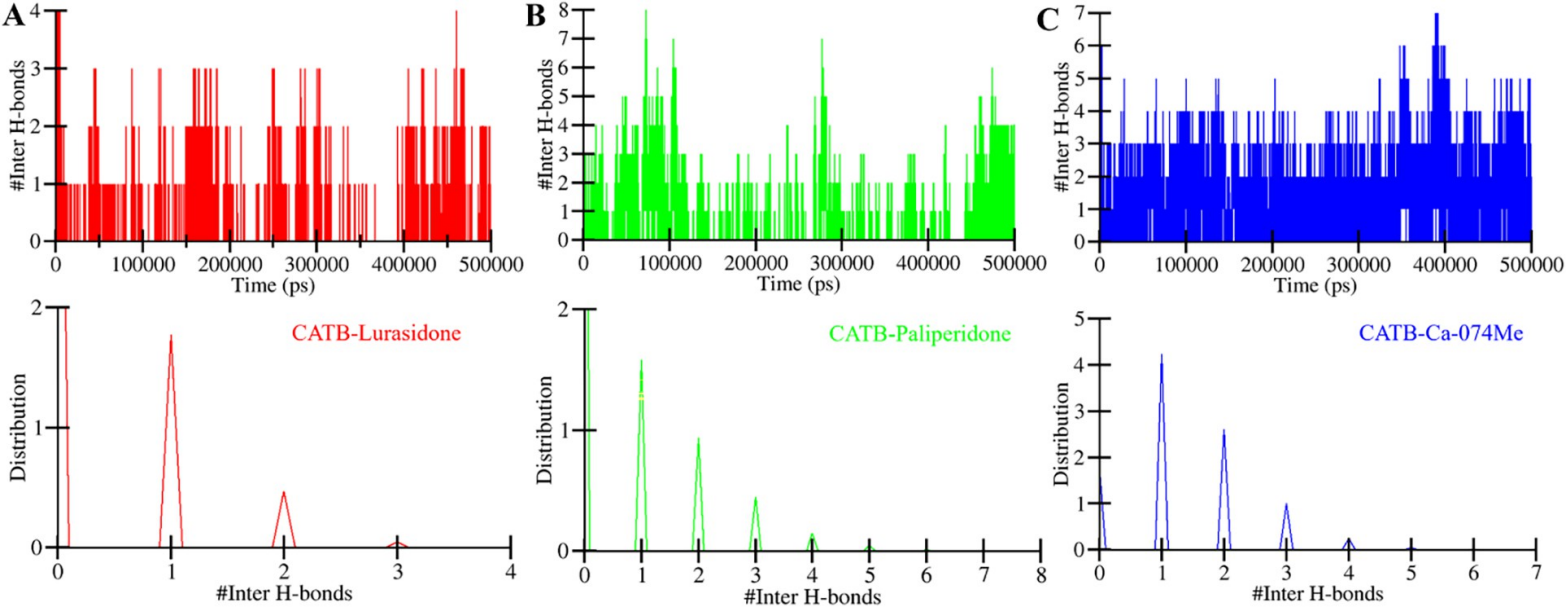
Intermolecular hydrogen bonds between CatB and (**A**) Lurasidone, (**B**) Paliperidone, and (**C**) Ca-074Me.

In addition to intramolecular hydrogen bonds, the time evolution of intermolecular hydrogen bonding was evaluated to explore the stability of the docked CatB with Lurasidone, Paliperidone, and Ca-074Me. The presence of intermolecular hydrogen bonds between the protein and small molecules governs the formation of stable docked complexes [53]. The number of hydrogen bonds formed in all three complexes was calculated to be 1–8 (**Fig 6, upper panel**). Lurasidone showed 1–4 hydrogen bonds with CatB, with higher stability of 1–2 bonds (**Fig 6A, upper panel**). At the same time, Paliperidone showed 1–8 hydrogen bonds with CatB, with higher stability of 2–3 bonds (**Fig 6B, upper panel**). When comparing with the reference inhibitor Ca-074Me, it showed 1–7 hydrogen bonds with CatB, with a higher stability of 1–3 bonds (**Fig 6C, upper panel**). The observed results indicate that Lurasidone, Paliperidone, and Ca-074Me remained in their original docking positions on CatB throughout the simulation, with a stable network of intermolecular hydrogen bonds contributing to their consistent interaction. Notably, the PDF plot showed consistently high probabilities for at least 1 hydrogen bond formation in all three complexes (**Fig 6, lower panel**). The increased PDF scores further support the idea that these complexes held their structural integrity and did not experience significant changes in their docking positions. Taken together, the intermolecular hydrogen bonding analysis reinforces the stability and reliability of the docked complexes suggesting that all the ligands effectively interact and remain bound to CatB throughout the simulation period.

#### 3.4.4. Principal component analysis

**Fig 7:**
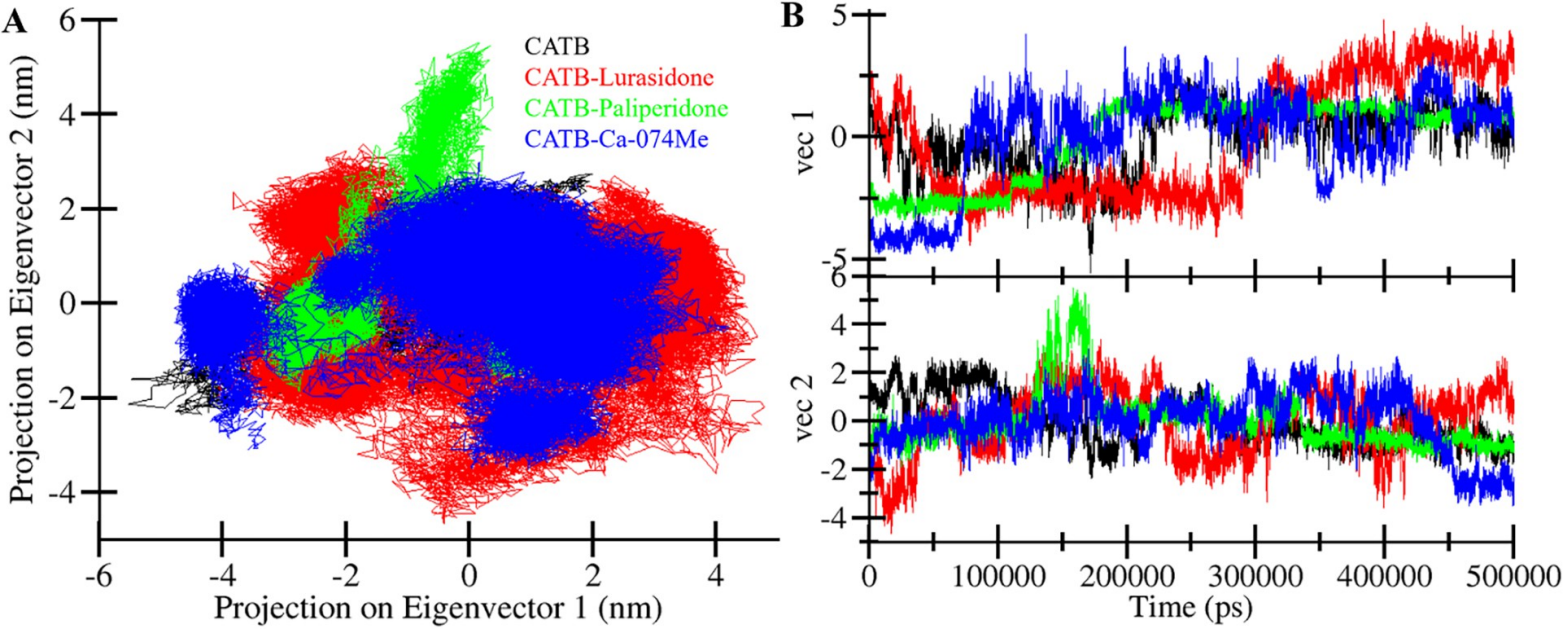
Conformational projection in principal component analysis. (**A**) 2D projection of CatB, CatB-Lurasidone, CatB-Paliperidone, and CatB-Ca-074Me. (**B**) Time evolution of the trajectories.

PCA is a powerful technique that helps in understanding the essential motions and conformational changes within biomolecular systems by identifying the major modes of motion present in the simulation data [40, 41]. This study employed PCA to explore the collective motions of CatB, CatB-Lurasidone, CatB-Paliperidone, and CATB-Ca-074Me. We computed the covariance matrix from the MD trajectory. The average trace of this matrix represents the total variance of atomic motions. CatB in the free state traced for −5 to 3 on EV1 and −2 to 2 on EV2. At the same time, CatB in the presence of Lurasidone, Paliperidone, and Ca-074Me traced for −4 to 4, −4 to 2, and −5 to 3 on EV1 and −4 to 3, −2 to 5, and −3 to 2 on EV2. This value provides a quantitative measure of the overall flexibility and conformational diversity observed in our simulated protein and protein systems. **Fig 7** illustrates the conformational sampling in the essential subspace for all four systems: CatB, CatB-Lurasidone, CatB-Paliperidone, and CATB-Ca-074Me. Remarkably, all the ligand-bound complexes were found to occupy almost the same essential subspace as the free CatB (**Fig 7A**). In both eigenvectors (EVs), the CATB-Paliperidone complex exhibited a subspace that closely resembled that of free CatB (**Fig 7B**). It displayed lower flexibility than the CATB-Lurasidone complex in both EVs, corroborating the findings from the earlier analyses as well. The close resemblance of the essential subspace between the free CatB and the CATB-Paliperidone complex suggests a certain level of conformational similarity, while the differences observed between CatB-Lurasidone and CATB-Ca-074Me complexes hint at distinct dynamic behaviors. This analysis enriches our understanding of the conformational dynamics of CatB and its interactions with Lurasidone, Paliperidone, and Ca-074Me, contributing to the overall characterization of these systems.

#### 3.4.5. Free energy landscapes analysis

**Fig 8:**
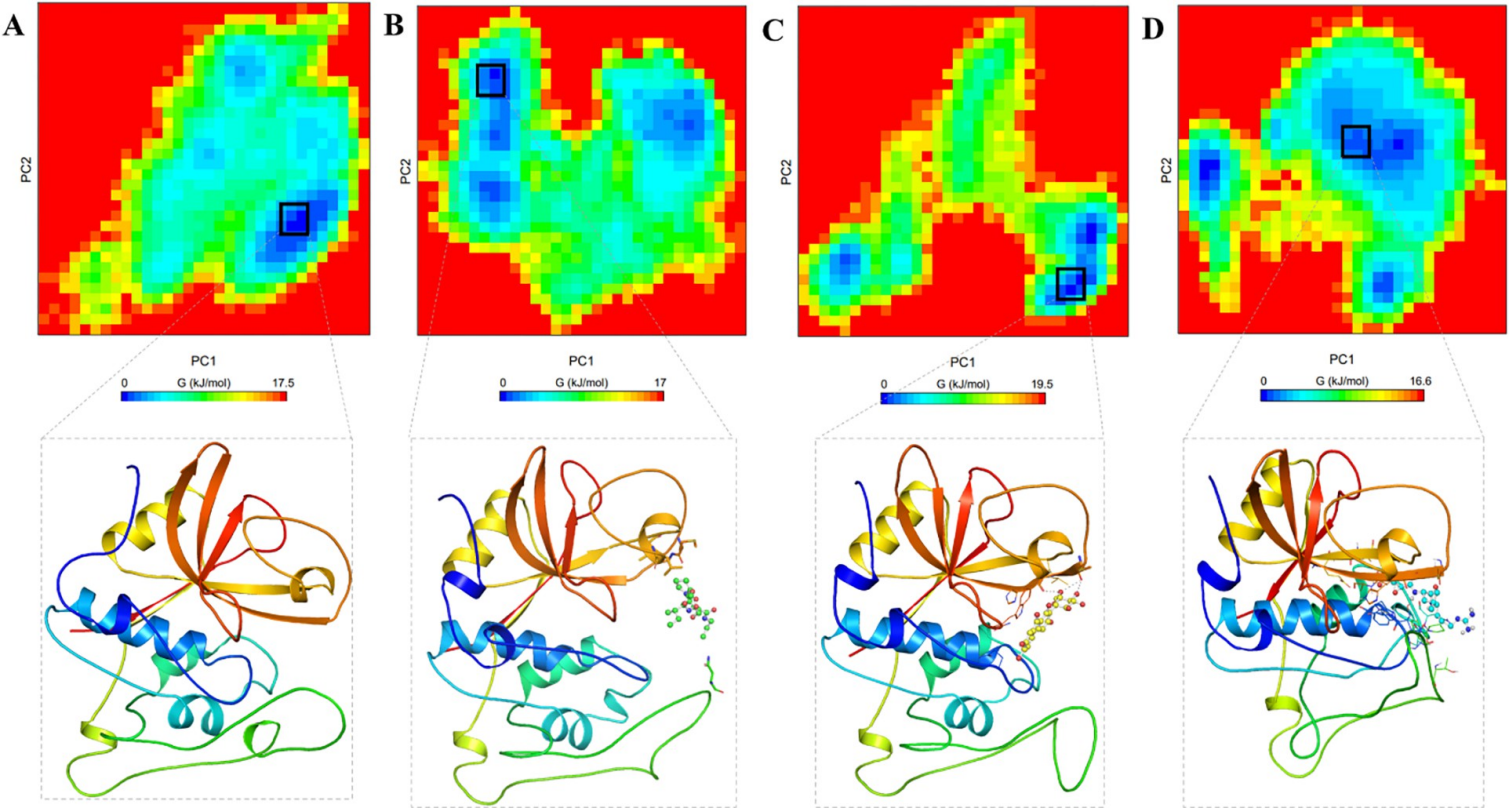
Free energy landscapes (FELs) plots of (**A**) free CatB, (**B**) CatB-Lurasidone, (**C**) CatB-Paliperidone, and (**D**) free CATB-Ca-074Me. Lower panels showed the most stable conformations of CatB, and its docked complexes fetched from the global minimum.

Free energy landscapes have become instrumental in describing protein folding mechanisms and assessing their stability in solvents, utilizing simulated trajectories [40]. In this study, the energy minima and conformational landscapes of CatB, CatB-Lurasidone, CatB-Paliperidone, and CatB-Ca-074Me complexes were examined using two principal components (PCs). **Fig 8** presents the contoured maps for the FELs of CatB, CatB-Lurasidone, CatB-Paliperidone, and CatB-Ca-074Me complexes. In the FELs, darker blue regions indicate conformations with lower energy near the native states. The plot of CatB illustrates that the size and position of the phases are restricted within a single stable global minimum (**Fig 8A**). According to the plots, CatB appears constrained within a single global minimum, which has expanded to 2–3 basins. On the other hand, CatB-Lurasidone, CatB-Paliperidone, and CATB-Ca-074Me exhibit different states, characterized by a lower global minimum and 2–3 local basins with varying populations (**Fig 8B–8D**). The most stable conformations of CatB and its docked complexes do not show any significant conformational alterations, as fetched from the global minimum (**Fig 8, lower panels**). Overall, the essential dynamics in PCA of CatB in complex with Lurasidone, Paliperidone, and Ca-074Me reveal conformational stability with some positional switching. The FEL analysis provides a comprehensive perspective on these complexes’ energy landscapes, which offers insights into their structural stability and dynamics during the simulation period.

### 3.5. MM-PBSA analysis

The MM-PBSA analysis gave information on the binding free energy of CatB to Lurasidone, Paliperidone, and Ca-074Me. The binding free energy components, including van der Waals force and its average standard deviation complexes obtained from the MMPBSA analysis, are depicted in **Table 4**. The binding free energies of the complexes were all negative, which suggests that the binding interactions are favorable. Among the studied compounds, Paliperidone had the most favorable binding energy at −38.82 kJ/mol, which indicates that it interacts most favorably with CatB. Lurasidone exhibited the second-highest binding affinity with a binding free energy of −26.44 kJ/mol, and Ca-074Me had the least favorable binding energy of −22.58 kJ/mol but still a negative value. The negative binding energies were mainly due to electrostatic forces, non-polar solvation energy and Van der Waals forces. On the other hand, polar solvation energy was positively correlated with the overall binding energy. This equilibrium of forces points to the fact that protein-ligand interactions are not always straightforward and that several factors must be considered when designing drugs. Based on these results, Paliperidone and Lurasidone can be considered as potential inhibitor candidates with high binding affinity towards CatB. The congruency of these results with other simulation analyses lends credibility to our findings and the prospect of these compounds as CatB inhibitors. Additional experimental work would be required to substantiate the observed inhibitory properties of these compounds on CatB activity. Furthermore, the study of the molecular interactions that make up the binding energies might be useful in further drug design studies aimed at CatB.

**Table 4:** Different binding parameters between the KatB-ligand complexes estimated in MM-PBSA calculations.

*Complex*	Δ*vdWaals*	*ΔE*_*EL*_	*ΔE*_*PB*_	*ΔE*_*NPOLAR*_	*ΔG*_*GAS*_	*ΔG*_*SOLV*_	*Standard deviation*	${\Delta G}_{Total}(kJ/mol)$
KatB-Lurasidone	−25.58	−92.10	103.02	−3.10	−112.72	106.41	4.15	−26.44
KatB-Paliperidone	−24.52	−108.02	54.88	−3.14	−130.58	108.04	3.32	−38.82
KatB-Ca-074Me	−30.82	−56.94	27.98	−3.41	−48.82	46.28	2.72	−22.58

## 4. Conclusions

CatB plays a critical role in AD development and cancer progression, making it a viable therapeutic target. This study identified Lurasidone and Paliperidone as effective CatB modulators through molecular docking and MD simulations. Both compounds demonstrated high binding affinities to functionally relevant residues in the CatB catalytic pocket, supporting their selectivity and therapeutic potential. Structural stability observed during MD simulations, along with insights from PCA and FEL analyses, further validated their potential as CatB inhibitors. However, this study has certain limitations, as the findings were derived from *in silico* analyses. Further *in vitro* and *in vivo* studies are necessary to confirm their efficacy. Future research could explore related proteases such as CatL and CatS or combine these repurposed drugs with conventional treatments to enhance therapeutic outcomes. Despite these limitations, Lurasidone and Paliperidone provide a strong foundation for repurposing as lead compounds for AD and cancer therapies.

## Supporting information

S1 Fig Chemical structures of CA-074Me, E-64, and selected synthesized molecules as CatB inhibitors. (DOCX)

S2 Fig Re-docking of a co-crystalized dipeptidyl nitrile inhibitor of CatB showing superimposition to each other. The figure was generated through PyMOL using the Protein Data Bank coordinates with ID: 1GMY.
(DOCX)
