# Supplementary figures and images for: Unveiling Cathepsin B inhibition with repurposed drugs for anticancer and anti-Alzheimer’s drug discovery
Source: PLoS One. 2024 Dec 19;19(12):e0316010. doi: 10.1371/journal.pone.0316010 (PMC11658610; doi:10.1371/journal.pone.0316010)

**
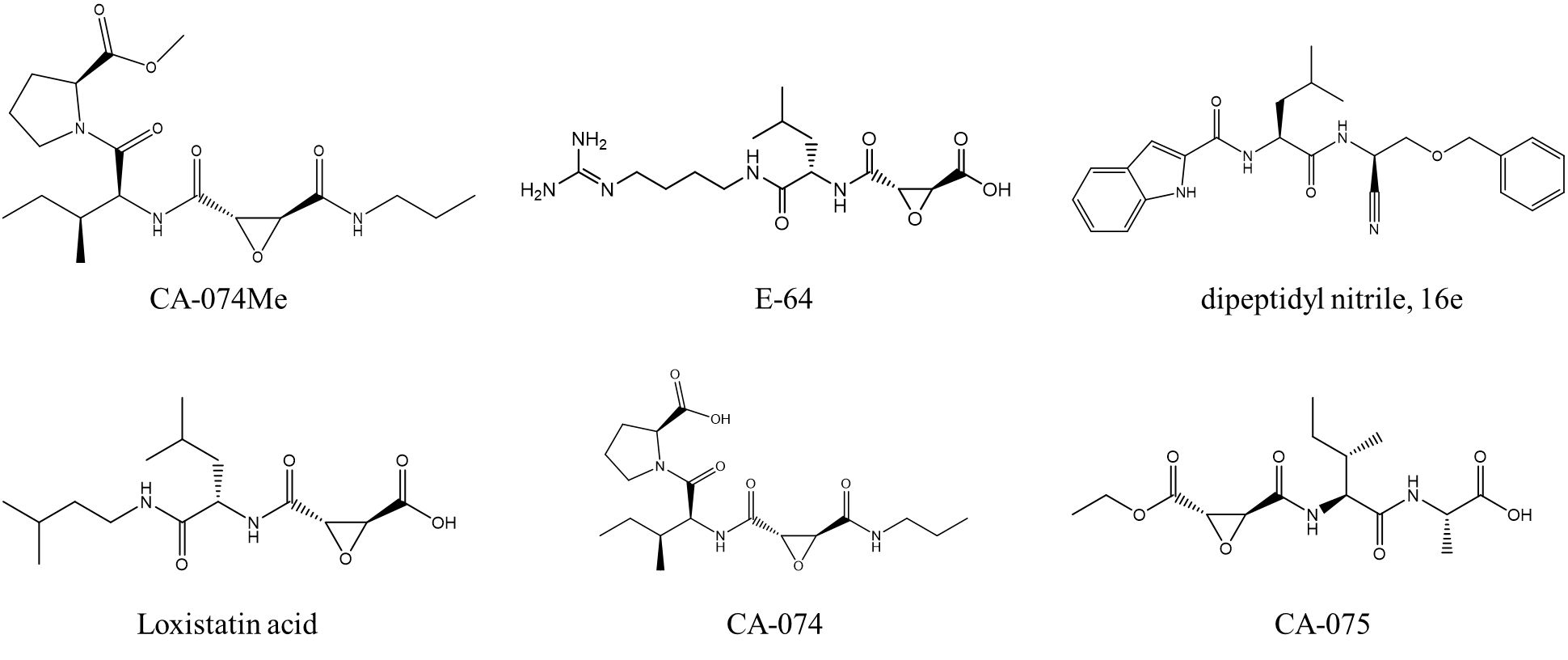
**

**Figure S1:** Chemical structures of CA-074Me, E-64, and selected synthesized molecules as CatB inhibitors.

Supplement: S1 Fig — (DOCX) [file pone.0316010.s001.docx]
