# Supplementary material for: Unveiling Cathepsin B inhibition with repurposed drugs for anticancer and anti-Alzheimer’s drug discovery
Source: PLoS One. 2024 Dec 19;19(12):e0316010. doi: 10.1371/journal.pone.0316010 (PMC11658610; doi:10.1371/journal.pone.0316010)
Supplement: S2 Fig — The figure was generated through PyMOL using the Protein Data Bank coordinates with ID: 1GMY. (DOCX) [file pone.0316010.s002.docx]

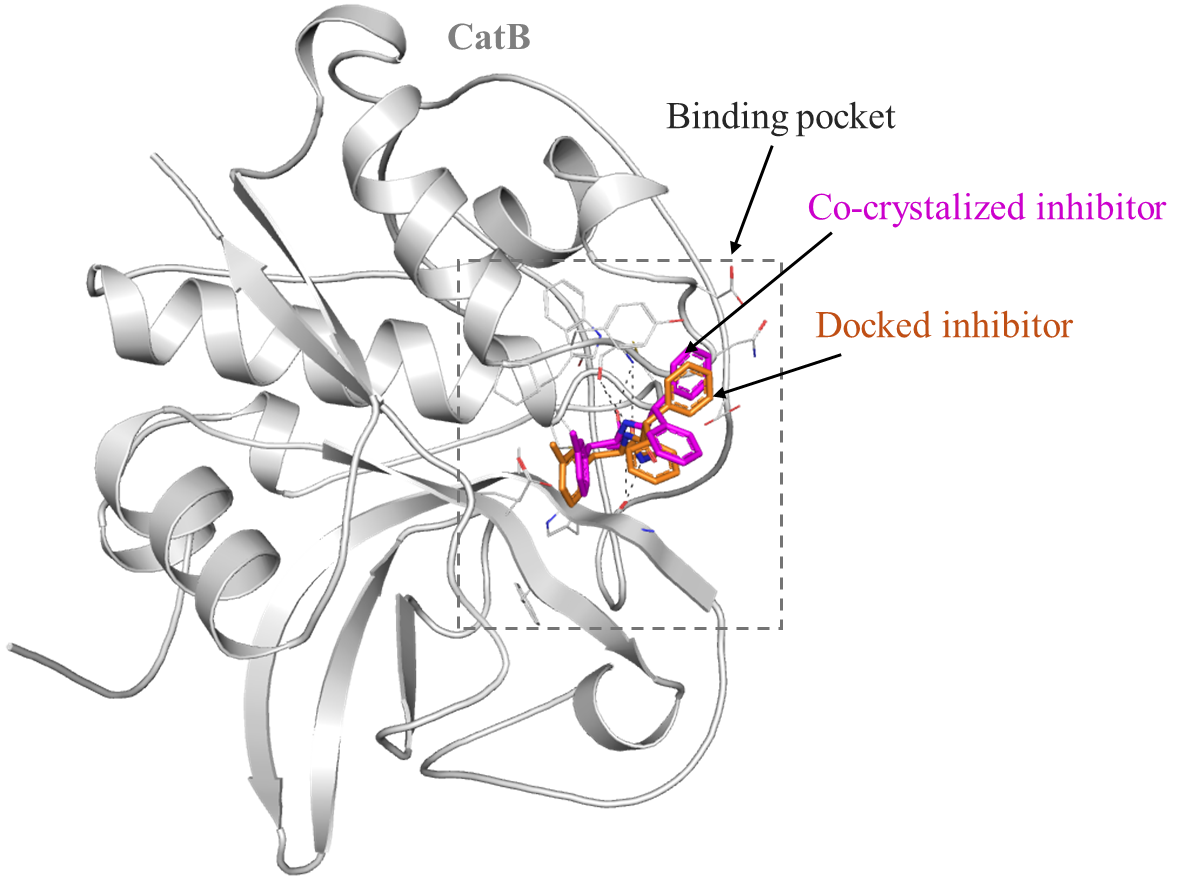


**Figure S2:** Re-docking of a co-crystalized dipeptidyl nitrile inhibitor of CatB showing superimposition to each other. The figure was generated through PyMOL using the Protein Data Bank coordinates with ID: 1GMY.
